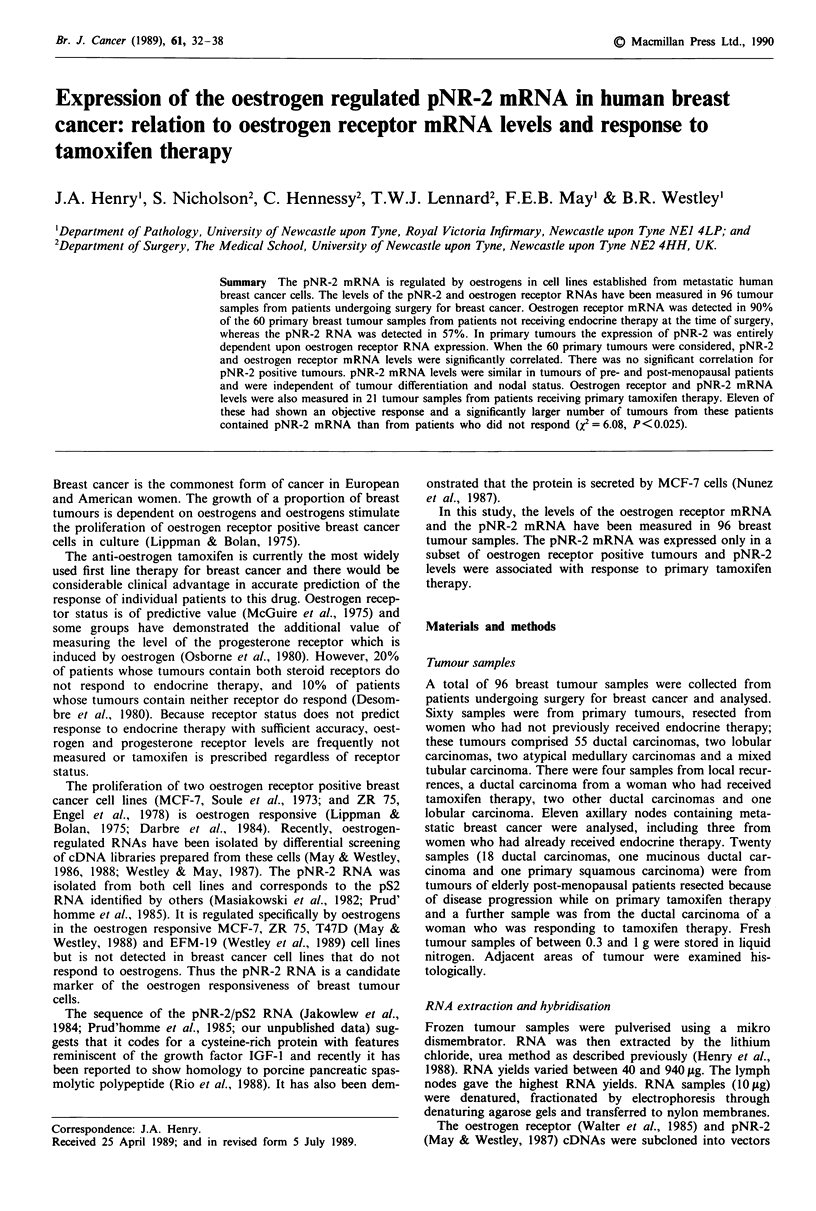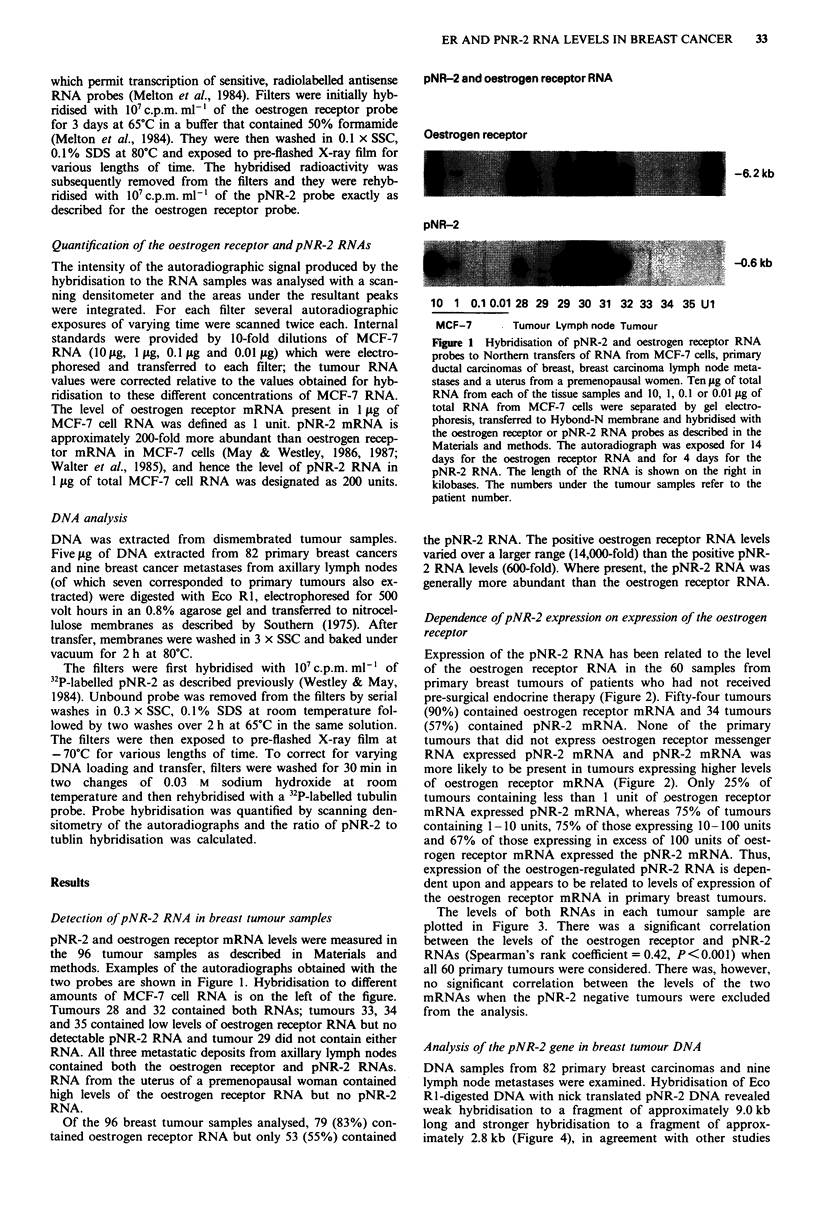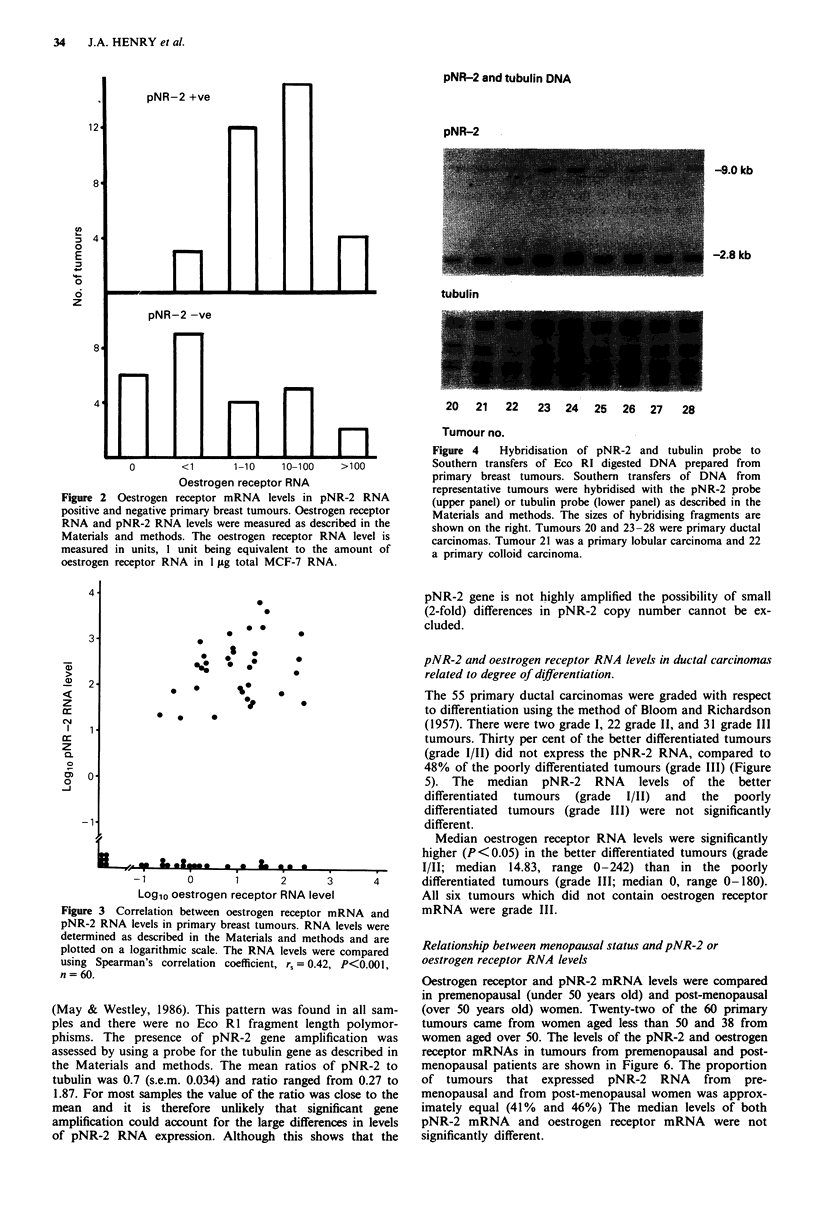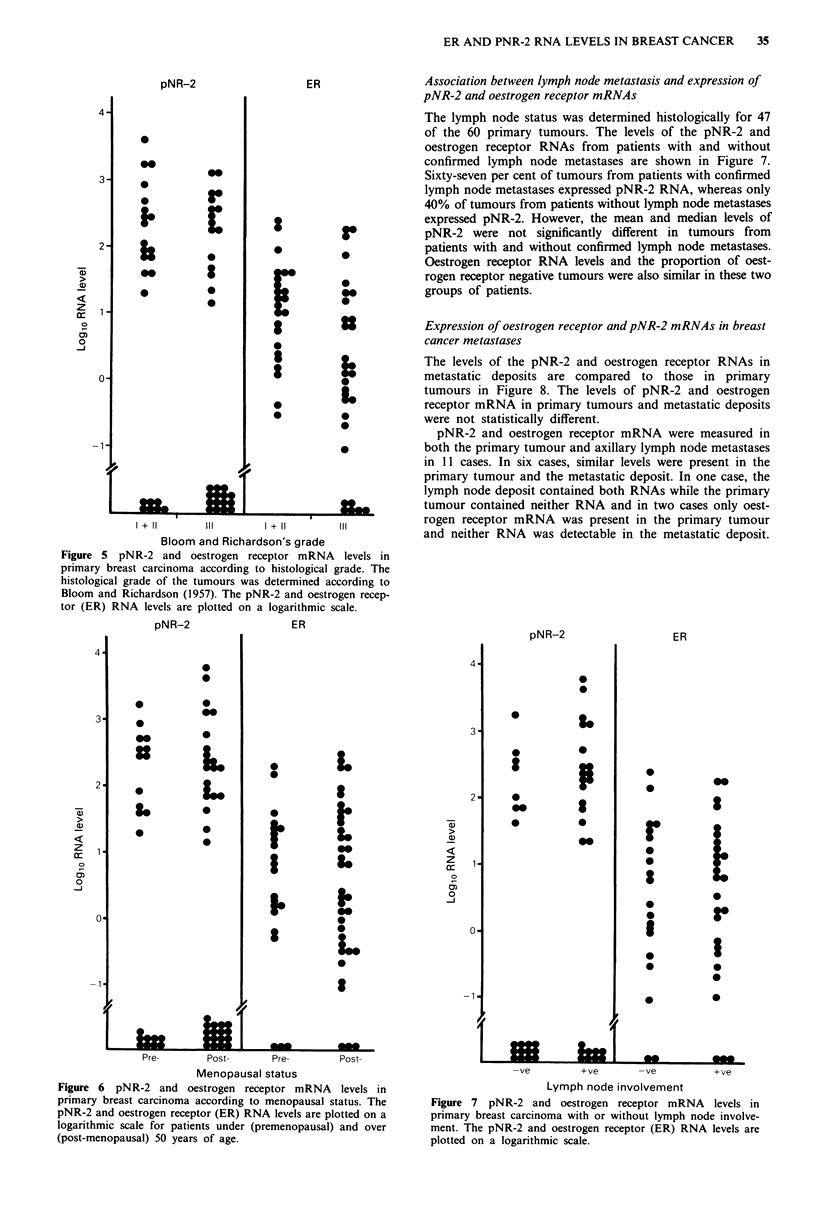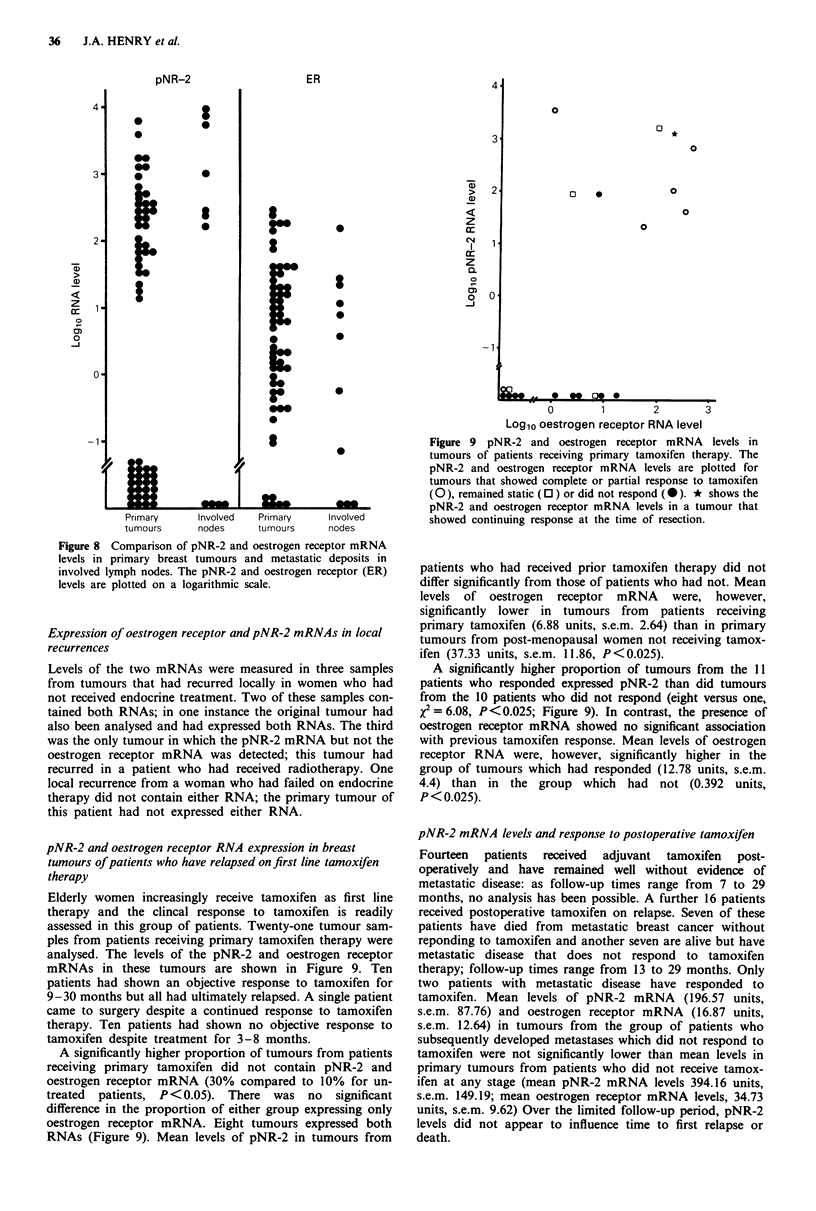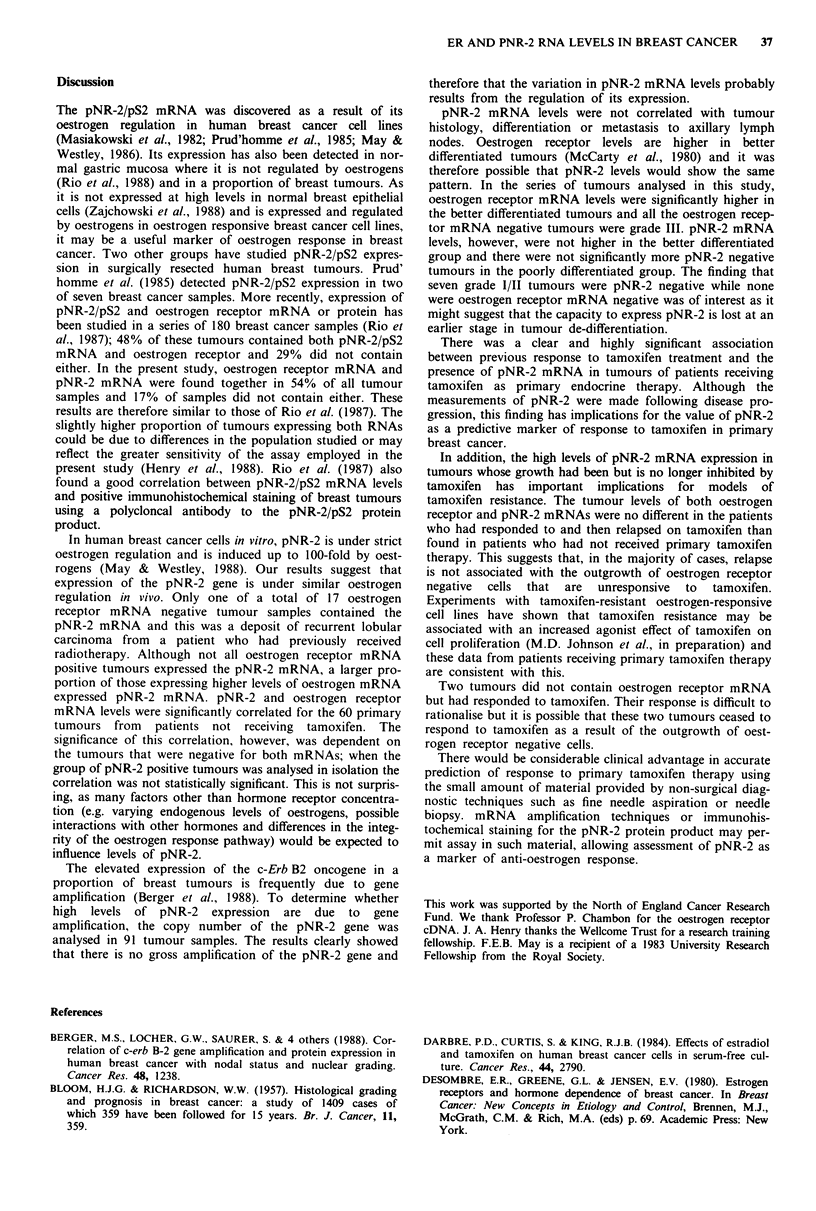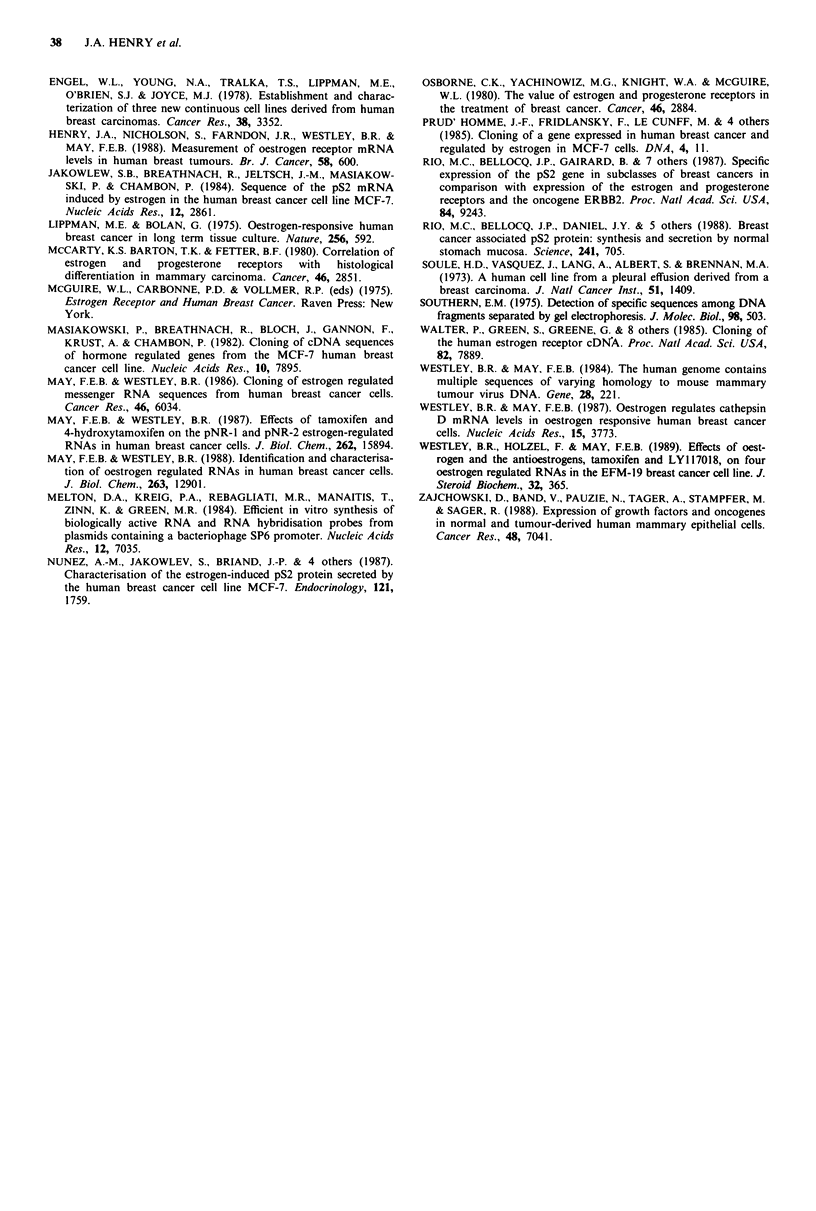# Expression of the oestrogen regulated pNR-2 mRNA in human breast cancer: relation to oestrogen receptor mRNA levels and response to tamoxifen therapy.

**DOI:** 10.1038/bjc.1990.8

**Published:** 1990-01

**Authors:** J. A. Henry, S. Nicholson, C. Hennessy, T. W. Lennard, F. E. May, B. R. Westley

**Affiliations:** Department of Pathology, University of Newcastle upon Tyne, Royal Victoria Infirmary, UK.

## Abstract

**Images:**


 © Macmillan Press Ltd., 1990

### Expression of the oestrogen regulated pNR-2 mRNA in human breast cancer: relation to oestrogen receptor mRNA levels and response to tamoxifen therapy

J.A. Henry[1], S. Nicholson[2], C. Hennessy[2], T.W.J. Lennard[2], F.E.B. May[1] & B.R. Westley[1]

[1]*Department of Pathology, University of Newcastle upon Tyne, Royal Victoria Infirmary, Newcastle upon Tyne NE1 4LP; and* [2]*Department of Surgery, The Medical School, University of Newcastle upon Tyne, Newcastle upon Tyne NE2 4HH, UK.*

**Summary** The pNR-2 mRNA is regulated by oestrogens in cell lines established from metastatic human breast cancer cells. The levels of the pNR-2 and oestrogen receptor RNAs have been measured in 96 tumour samples from patients undergoing surgery for breast cancer. Oestrogen receptor mRNA was detected in 90% of the 60 primary breast tumour samples from patients not receiving endocrine therapy at the time of surgery, whereas the pNR-2 RNA was detected in 57%. In primary tumours the expression of pNR-2 was entirely dependent upon oestrogen receptor RNA expression. When the 60 primary tumours were considered, pNR-2 and oestrogen receptor mRNA levels were significantly correlated. There was no significant correlation for pNR-2 positive tumours. pNR-2 mRNA levels were similar in tumours of pre- and post-menopausal patients and were independent of tumour differentiation and nodal status. Oestrogen receptor and pNR-2 mRNA levels were also measured in 21 tumour samples from patients receiving primary tamoxifen therapy. Eleven of these had shown an objective response and a significantly larger number of tumours from these patients contained pNR-2 mRNA than from patients who did not respond ($\chi^2 = 6.08$, $P < 0.025$).

Breast cancer is the commonest form of cancer in European and American women. The growth of a proportion of breast tumours is dependent on oestrogens and oestrogens stimulate the proliferation of oestrogen receptor positive breast cancer cells in culture (Lippman & Bolan, 1975).

The anti-oestrogen tamoxifen is currently the most widely used first line therapy for breast cancer and there would be considerable clinical advantage in accurate prediction of the response of individual patients to this drug. Oestrogen receptor status is of predictive value (McGuire *et al.*, 1975) and some groups have demonstrated the additional value of measuring the level of the progesterone receptor which is induced by oestrogen (Osborne *et al.*, 1980). However, 20% of patients whose tumours contain both steroid receptors do not respond to endocrine therapy, and 10% of patients whose tumours contain neither receptor do respond (Desombre *et al.*, 1980). Because receptor status does not predict response to endocrine therapy with sufficient accuracy, oestrogen and progesterone receptor levels are frequently not measured or tamoxifen is prescribed regardless of receptor status.

The proliferation of two oestrogen receptor positive breast cancer cell lines (MCF-7, Soule *et al.*, 1973; and ZR 75, Engel *et al.*, 1978) is oestrogen responsive (Lippman & Bolan, 1975; Darbre *et al.*, 1984). Recently, oestrogen-regulated RNAs have been isolated by differential screening of cDNA libraries prepared from these cells (May & Westley, 1986, 1988; Westley & May, 1987). The pNR-2 RNA was isolated from both cell lines and corresponds to the pS2 RNA identified by others (Masiakowski *et al.*, 1982; Prud' homme *et al.*, 1985). It is regulated specifically by oestrogens in the oestrogen responsive MCF-7, ZR 75, T47D (May & Westley, 1988) and EFM-19 (Westley *et al.*, 1989) cell lines but is not detected in breast cancer cell lines that do not respond to oestrogens. Thus the pNR-2 RNA is a candidate marker of the oestrogen responsiveness of breast tumour cells.

The sequence of the pNR-2/pS2 RNA (Jakowlew *et al.*, 1984; Prud'homme *et al.*, 1985; our unpublished data) suggests that it codes for a cysteine-rich protein with features reminiscent of the growth factor IGF-1 and recently it has been reported to show homology to porcine pancreatic spasmolytic polypeptide (Rio *et al.*, 1988). It has also been demonstrated that the protein is secreted by MCF-7 cells (Nunez *et al.*, 1987).

Correspondence: J.A. Henry.
Received 25 April 1989; and in revised form 5 July 1989.

In this study, the levels of the oestrogen receptor mRNA and the pNR-2 mRNA have been measured in 96 breast tumour samples. The pNR-2 mRNA was expressed only in a subset of oestrogen receptor positive tumours and pNR-2 levels were associated with response to primary tamoxifen therapy.

### Materials and methods

### *Tumour samples*

A total of 96 breast tumour samples were collected from patients undergoing surgery for breast cancer and analysed. Sixty samples were from primary tumours, resected from women who had not previously received endocrine therapy; these tumours comprised 55 ductal carcinomas, two lobular carcinomas, two atypical medullary carcinomas and a mixed tubular carcinoma. There were four samples from local recurrences, a ductal carcinoma from a woman who had received tamoxifen therapy, two other ductal carcinomas and one lobular carcinoma. Eleven axillary nodes containing metastatic breast cancer were analysed, including three from women who had already received endocrine therapy. Twenty samples (18 ductal carcinomas, one mucinous ductal carcinoma and one primary squamous carcinoma) were from tumours of elderly post-menopausal patients resected because of disease progression while on primary tamoxifen therapy and a further sample was from the ductal carcinoma of a woman who was responding to tamoxifen therapy. Fresh tumour samples of between 0.3 and 1 g were stored in liquid nitrogen. Adjacent areas of tumour were examined histologically.

### *RNA extraction and hybridisation*

Frozen tumour samples were pulverised using a mikro dismembrator. RNA was then extracted by the lithium chloride, urea method as described previously (Henry *et al.*, 1988). RNA yields varied between 40 and 940 μg. The lymph nodes gave the highest RNA yields. RNA samples (10 μg) were denatured, fractionated by electrophoresis through denaturing agarose gels and transferred to nylon membranes.

The oestrogen receptor (Walter *et al.*, 1985) and pNR-2 (May & Westley, 1987) cDNAs were subcloned into vectors which permit transcription of sensitive, radiolabelled antisense RNA probes (Melton *et al.*, 1984). Filters were initially hybridised with $10^7$ c.p.m. $ml^{-1}$ of the oestrogen receptor probe for 3 days at 65°C in a buffer that contained 50% formamide (Melton *et al.*, 1984). They were then washed in 0.1 × SSC, 0.1% SDS at 80°C and exposed to pre-flashed X-ray film for various lengths of time. The hybridised radioactivity was subsequently removed from the filters and they were rehybridised with $10^7$ c.p.m. $ml^{-1}$ of the pNR-2 probe exactly as described for the oestrogen receptor probe.

*Quantification of the oestrogen receptor and pNR-2 RNAs*

The intensity of the autoradiographic signal produced by the hybridisation to the RNA samples was analysed with a scanning densitometer and the areas under the resultant peaks were integrated. For each filter several autoradiographic exposures of varying time were scanned twice each. Internal standards were provided by 10-fold dilutions of MCF-7 RNA (10 μg, 1 μg, 0.1 μg and 0.01 μg) which were electrophoresed and transferred to each filter; the tumour RNA values were corrected relative to the values obtained for hybridisation to these different concentrations of MCF-7 RNA. The level of oestrogen receptor mRNA present in 1 μg of MCF-7 cell RNA was defined as 1 unit. pNR-2 mRNA is approximately 200-fold more abundant than oestrogen receptor mRNA in MCF-7 cells (May & Westley, 1986, 1987; Walter *et al.*, 1985), and hence the level of pNR-2 RNA in 1 μg of total MCF-7 cell RNA was designated as 200 units.

*DNA analysis*

DNA was extracted from dismembrated tumour samples. Five μg of DNA extracted from 82 primary breast cancers and nine breast cancer metastases from axillary lymph nodes (of which seven corresponded to primary tumours also extracted) were digested with Eco R1, electrophoresed for 500 volt hours in an 0.8% agarose gel and transferred to nitrocellulose membranes as described by Southern (1975). After transfer, membranes were washed in 3 × SSC and baked under vacuum for 2 h at 80°C.

The filters were first hybridised with $10^7$ c.p.m. $ml^{-1}$ of $^{32}$P-labelled pNR-2 as described previously (Westley & May, 1984). Unbound probe was removed from the filters by serial washes in 0.3 × SSC, 0.1% SDS at room temperature followed by two washes over 2 h at 65°C in the same solution. The filters were then exposed to pre-flashed X-ray film at −70°C for various lengths of time. To correct for varying DNA loading and transfer, filters were washed for 30 min in two changes of 0.03 M sodium hydroxide at room temperature and then rehybridised with a $^{32}$P-labelled tubulin probe. Probe hybridisation was quantified by scanning densitometry of the autoradiographs and the ratio of pNR-2 to tublin hybridisation was calculated.

### Results

*Detection of pNR-2 RNA in breast tumour samples*

pNR-2 and oestrogen receptor mRNA levels were measured in the 96 tumour samples as described in Materials and methods. Examples of the autoradiographs obtained with the two probes are shown in Figure 1. Hybridisation to different amounts of MCF-7 cell RNA is on the left of the figure. Tumours 28 and 32 contained both RNAs; tumours 33, 34 and 35 contained low levels of oestrogen receptor RNA but no detectable pNR-2 RNA and tumour 29 did not contain either RNA. All three metastatic deposits from axillary lymph nodes contained both the oestrogen receptor and pNR-2 RNAs. RNA from the uterus of a premenopausal woman contained high levels of the oestrogen receptor RNA but no pNR-2 RNA.

Of the 96 breast tumour samples analysed, 79 (83%) contained oestrogen receptor RNA but only 53 (55%) contained

**Figure 1** Hybridisation of pNR-2 and oestrogen receptor RNA probes to Northern transfers of RNA from MCF-7 cells, primary ductal carcinomas of breast, breast carcinoma lymph node metastases and a uterus from a premenopausal women. Ten μg of total RNA from each of the tissue samples and 10, 1, 0.1 or 0.01 μg of total RNA from MCF-7 cells were separated by gel electrophoresis, transferred to Hybond-N membrane and hybridised with the oestrogen receptor or pNR-2 RNA probes as described in the Materials and methods. The autoradiograph was exposed for 14 days for the oestrogen receptor RNA and for 4 days for the pNR-2 RNA. The length of the RNA is shown on the right in kilobases. The numbers under the tumour samples refer to the patient number.

the pNR-2 RNA. The positive oestrogen receptor RNA levels varied over a larger range (14,000-fold) than the positive pNR-2 RNA levels (600-fold). Where present, the pNR-2 RNA was generally more abundant than the oestrogen receptor RNA.

*Dependence of pNR-2 expression on expression of the oestrogen receptor*

Expression of the pNR-2 RNA has been related to the level of the oestrogen receptor RNA in the 60 samples from primary breast tumours of patients who had not received pre-surgical endocrine therapy (Figure 2). Fifty-four tumours (90%) contained oestrogen receptor mRNA and 34 tumours (57%) contained pNR-2 mRNA. None of the primary tumours that did not express oestrogen receptor messenger RNA expressed pNR-2 mRNA and pNR-2 mRNA was more likely to be present in tumours expressing higher levels of oestrogen receptor mRNA (Figure 2). Only 25% of tumours containing less than 1 unit of oestrogen receptor mRNA expressed pNR-2 mRNA, whereas 75% of tumours containing 1–10 units, 75% of those expressing 10–100 units and 67% of those expressing in excess of 100 units of oestrogen receptor mRNA expressed the pNR-2 mRNA. Thus, expression of the oestrogen-regulated pNR-2 RNA is dependent upon and appears to be related to levels of expression of the oestrogen receptor mRNA in primary breast tumours.

The levels of both RNAs in each tumour sample are plotted in Figure 3. There was a significant correlation between the levels of the oestrogen receptor and pNR-2 RNAs (Spearman's rank coefficient = 0.42, $P < 0.001$) when all 60 primary tumours were considered. There was, however, no significant correlation between the levels of the two mRNAs when the pNR-2 negative tumours were excluded from the analysis.

*Analysis of the pNR-2 gene in breast tumour DNA*

DNA samples from 82 primary breast carcinomas and nine lymph node metastases were examined. Hybridisation of Eco R1-digested DNA with nick translated pNR-2 DNA revealed weak hybridisation to a fragment of approximately 9.0 kb long and stronger hybridisation to a fragment of approximately 2.8 kb (Figure 4), in agreement with other studies

**Figure 2** Oestrogen receptor mRNA levels in pNR-2 RNA positive and negative primary breast tumours. Oestrogen receptor RNA and pNR-2 RNA levels were measured as described in the Materials and methods. The oestrogen receptor RNA level is measured in units, 1 unit being equivalent to the amount of oestrogen receptor RNA in 1 μg total MCF-7 RNA.

**Figure 3** Correlation between oestrogen receptor mRNA and pNR-2 RNA levels in primary breast tumours. RNA levels were determined as described in the Materials and methods and are plotted on a logarithmic scale. The RNA levels were compared using Spearman's correlation coefficient, $r_s = 0.42$, $P<0.001$, $n = 60$.

**Figure 4** Hybridisation of pNR-2 and tubulin probe to Southern transfers of Eco RI digested DNA prepared from primary breast tumours. Southern transfers of DNA from representative tumours were hybridised with the pNR-2 probe (upper panel) or tubulin probe (lower panel) as described in the Materials and methods. The sizes of hybridising fragments are shown on the right. Tumours 20 and 23-28 were primary ductal carcinomas. Tumour 21 was a primary lobular carcinoma and 22 a primary colloid carcinoma.

(May & Westley, 1986). This pattern was found in all samples and there were no Eco R1 fragment length polymorphisms. The presence of pNR-2 gene amplification was assessed by using a probe for the tubulin gene as described in the Materials and methods. The mean ratios of pNR-2 to tubulin was 0.7 (s.e.m. 0.034) and ratio ranged from 0.27 to 1.87. For most samples the value of the ratio was close to the mean and it is therefore unlikely that significant gene amplification could account for the large differences in levels of pNR-2 RNA expression. Although this shows that the pNR-2 gene is not highly amplified the possibility of small (2-fold) differences in pNR-2 copy number cannot be excluded.

*pNR-2 and oestrogen receptor RNA levels in ductal carcinomas related to degree of differentiation.*

The 55 primary ductal carcinomas were graded with respect to differentiation using the method of Bloom and Richardson (1957). There were two grade I, 22 grade II, and 31 grade III tumours. Thirty per cent of the better differentiated tumours (grade I/II) did not express the pNR-2 RNA, compared to 48% of the poorly differentiated tumours (grade III) (Figure 5). The median pNR-2 RNA levels of the better differentiated tumours (grade I/II) and the poorly differentiated tumours (grade III) were not significantly different.

Median oestrogen receptor RNA levels were significantly higher ($P<0.05$) in the better differentiated tumours (grade I/II; median 14.83, range 0-242) than in the poorly differentiated tumours (grade III; median 0, range 0-180). All six tumours which did not contain oestrogen receptor mRNA were grade III.

*Relationship between menopausal status and pNR-2 or oestrogen receptor RNA levels*

Oestrogen receptor and pNR-2 mRNA levels were compared in premenopausal (under 50 years old) and post-menopausal (over 50 years old) women. Twenty-two of the 60 primary tumours came from women aged less than 50 and 38 from women aged over 50. The levels of the pNR-2 and oestrogen receptor mRNAs in tumours from premenopausal and post-menopausal patients are shown in Figure 6. The proportion of tumours that expressed pNR-2 RNA from pre-menopausal and from post-menopausal women was approximately equal (41% and 46%) The median levels of both pNR-2 mRNA and oestrogen receptor mRNA were not significantly different.

**Figure 5** pNR-2 and oestrogen receptor mRNA levels in primary breast carcinoma according to histological grade. The histological grade of the tumours was determined according to Bloom and Richardson (1957). The pNR-2 and oestrogen receptor (ER) RNA levels are plotted on a logarithmic scale.

**Figure 6** pNR-2 and oestrogen receptor mRNA levels in primary breast carcinoma according to menopausal status. The pNR-2 and oestrogen receptor (ER) RNA levels are plotted on a logarithmic scale for patients under (premenopausal) and over (post-menopausal) 50 years of age.

*Association between lymph node metastasis and expression of pNR-2 and oestrogen receptor mRNAs*

The lymph node status was determined histologically for 47 of the 60 primary tumours. The levels of the pNR-2 and oestrogen receptor RNAs from patients with and without confirmed lymph node metastases are shown in Figure 7. Sixty-seven per cent of tumours from patients with confirmed lymph node metastases expressed pNR-2 RNA, whereas only 40% of tumours from patients without lymph node metastases expressed pNR-2. However, the mean and median levels of pNR-2 were not significantly different in tumours from patients with and without confirmed lymph node metastases. Oestrogen receptor RNA levels and the proportion of oestrogen receptor negative tumours were also similar in these two groups of patients.

*Expression of oestrogen receptor and pNR-2 mRNAs in breast cancer metastases*

The levels of the pNR-2 and oestrogen receptor RNAs in metastatic deposits are compared to those in primary tumours in Figure 8. The levels of pNR-2 and oestrogen receptor mRNA in primary tumours and metastatic deposits were not statistically different.

pNR-2 and oestrogen receptor mRNA were measured in both the primary tumour and axillary lymph node metastases in 11 cases. In six cases, similar levels were present in the primary tumour and the metastatic deposit. In one case, the lymph node deposit contained both RNAs while the primary tumour contained neither RNA and in two cases only oestrogen receptor mRNA was present in the primary tumour and neither RNA was detectable in the metastatic deposit.

**Figure 7** pNR-2 and oestrogen receptor mRNA levels in primary breast carcinoma with or without lymph node involvement. The pNR-2 and oestrogen receptor (ER) RNA levels are plotted on a logarithmic scale.

**Figure 8** Comparison of pNR-2 and oestrogen receptor mRNA levels in primary breast tumours and metastatic deposits in involved lymph nodes. The pNR-2 and oestrogen receptor (ER) levels are plotted on a logarithmic scale.

**Figure 9** pNR-2 and oestrogen receptor mRNA levels in tumours of patients receiving primary tamoxifen therapy. The pNR-2 and oestrogen receptor mRNA levels are plotted for tumours that showed complete or partial response to tamoxifen (○), remained static (□) or did not respond (●). ★ shows the pNR-2 and oestrogen receptor mRNA levels in a tumour that showed continuing response at the time of resection.

*Expression of oestrogen receptor and pNR-2 mRNAs in local recurrences*

Levels of the two mRNAs were measured in three samples from tumours that had recurred locally in women who had not received endocrine treatment. Two of these samples contained both RNAs; in one instance the original tumour had also been analysed and had expressed both RNAs. The third was the only tumour in which the pNR-2 mRNA but not the oestrogen receptor mRNA was detected; this tumour had recurred in a patient who had received radiotherapy. One local recurrence from a woman who had failed on endocrine therapy did not contain either RNA; the primary tumour of this patient had not expressed either RNA.

*pNR-2 and oestrogen receptor RNA expression in breast tumours of patients who have relapsed on first line tamoxifen therapy*

Elderly women increasingly receive tamoxifen as first line therapy and the clincal response to tamoxifen is readily assessed in this group of patients. Twenty-one tumour samples from patients receiving primary tamoxifen therapy were analysed. The levels of the pNR-2 and oestrogen receptor mRNAs in these tumours are shown in Figure 9. Ten patients had shown an objective response to tamoxifen for 9–30 months but all had ultimately relapsed. A single patient came to surgery despite a continued response to tamoxifen therapy. Ten patients had shown no objective response to tamoxifen despite treatment for 3–8 months.

A significantly higher proportion of tumours from patients receiving primary tamoxifen did not contain pNR-2 and oestrogen receptor mRNA (30% compared to 10% for untreated patients, $P<0.05$). There was no significant difference in the proportion of either group expressing only oestrogen receptor mRNA. Eight tumours expressed both RNAs (Figure 9). Mean levels of pNR-2 in tumours from patients who had received prior tamoxifen therapy did not differ significantly from those of patients who had not. Mean levels of oestrogen receptor mRNA were, however, significantly lower in tumours from patients receiving primary tamoxifen (6.88 units, s.e.m. 2.64) than in primary tumours from post-menopausal women not receiving tamoxifen (37.33 units, s.e.m. 11.86, $P<0.025$).

A significantly higher proportion of tumours from the 11 patients who responded expressed pNR-2 than did tumours from the 10 patients who did not respond (eight versus one, $\chi^2 = 6.08$, $P<0.025$; Figure 9). In contrast, the presence of oestrogen receptor mRNA showed no significant association with previous tamoxifen response. Mean levels of oestrogen receptor RNA were, however, significantly higher in the group of tumours which had responded (12.78 units, s.e.m. 4.4) than in the group which had not (0.392 units, $P<0.025$).

*pNR-2 mRNA levels and response to postoperative tamoxifen*

Fourteen patients received adjuvant tamoxifen postoperatively and have remained well without evidence of metastatic disease: as follow-up times range from 7 to 29 months, no analysis has been possible. A further 16 patients received postoperative tamoxifen on relapse. Seven of these patients have died from metastatic breast cancer without reponding to tamoxifen and another seven are alive but have metastatic disease that does not respond to tamoxifen therapy; follow-up times range from 13 to 29 months. Only two patients with metastatic disease have responded to tamoxifen. Mean levels of pNR-2 mRNA (196.57 units, s.e.m. 87.76) and oestrogen receptor mRNA (16.87 units, s.e.m. 12.64) in tumours from the group of patients who subsequently developed metastases which did not respond to tamoxifen were not significantly lower than mean levels in primary tumours from patients who did not receive tamoxifen at any stage (mean pNR-2 mRNA levels 394.16 units, s.e.m. 149.19; mean oestrogen receptor mRNA levels, 34.73 units, s.e.m. 9.62) Over the limited follow-up period, pNR-2 levels did not appear to influence time to first relapse or death.

### Discussion

The pNR-2/pS2 mRNA was discovered as a result of its oestrogen regulation in human breast cancer cell lines (Masiakowski *et al.*, 1982; Prud'homme *et al.*, 1985; May & Westley, 1986). Its expression has also been detected in normal gastric mucosa where it is not regulated by oestrogens (Rio *et al.*, 1988) and in a proportion of breast tumours. As it is not expressed at high levels in normal breast epithelial cells (Zajchowski *et al.*, 1988) and is expressed and regulated by oestrogens in oestrogen responsive breast cancer cell lines, it may be a useful marker of oestrogen response in breast cancer. Two other groups have studied pNR-2/pS2 expression in surgically resected human breast tumours. Prud' homme *et al.* (1985) detected pNR-2/pS2 expression in two of seven breast cancer samples. More recently, expression of pNR-2/pS2 and oestrogen receptor mRNA or protein has been studied in a series of 180 breast cancer samples (Rio *et al.*, 1987); 48% of these tumours contained both pNR-2/pS2 mRNA and oestrogen receptor and 29% did not contain either. In the present study, oestrogen receptor mRNA and pNR-2 mRNA were found together in 54% of all tumour samples and 17% of samples did not contain either. These results are therefore similar to those of Rio *et al.* (1987). The slightly higher proportion of tumours expressing both RNAs could be due to differences in the population studied or may reflect the greater sensitivity of the assay employed in the present study (Henry *et al.*, 1988). Rio *et al.* (1987) also found a good correlation between pNR-2/pS2 mRNA levels and positive immunohistochemical staining of breast tumours using a polycloncal antibody to the pNR-2/pS2 protein product.

In human breast cancer cells *in vitro*, pNR-2 is under strict oestrogen regulation and is induced up to 100-fold by oestrogens (May & Westley, 1988). Our results suggest that expression of the pNR-2 gene is under similar oestrogen regulation *in vivo*. Only one of a total of 17 oestrogen receptor mRNA negative tumour samples contained the pNR-2 mRNA and this was a deposit of recurrent lobular carcinoma from a patient who had previously received radiotherapy. Although not all oestrogen receptor mRNA positive tumours expressed the pNR-2 mRNA, a larger proportion of those expressing higher levels of oestrogen mRNA expressed pNR-2 mRNA. pNR-2 and oestrogen receptor mRNA levels were significantly correlated for the 60 primary tumours from patients not receiving tamoxifen. The significance of this correlation, however, was dependent on the tumours that were negative for both mRNAs; when the group of pNR-2 positive tumours was analysed in isolation the correlation was not statistically significant. This is not surprising, as many factors other than hormone receptor concentration (e.g. varying endogenous levels of oestrogens, possible interactions with other hormones and differences in the integrity of the oestrogen response pathway) would be expected to influence levels of pNR-2.

The elevated expression of the c-*Erb* B2 oncogene in a proportion of breast tumours is frequently due to gene amplification (Berger *et al.*, 1988). To determine whether high levels of pNR-2 expression are due to gene amplification, the copy number of the pNR-2 gene was analysed in 91 tumour samples. The results clearly showed that there is no gross amplification of the pNR-2 gene and therefore that the variation in pNR-2 mRNA levels probably results from the regulation of its expression.

pNR-2 mRNA levels were not correlated with tumour histology, differentiation or metastasis to axillary lymph nodes. Oestrogen receptor levels are higher in better differentiated tumours (McCarty *et al.*, 1980) and it was therefore possible that pNR-2 levels would show the same pattern. In the series of tumours analysed in this study, oestrogen receptor mRNA levels were significantly higher in the better differentiated tumours and all the oestrogen receptor mRNA negative tumours were grade III. pNR-2 mRNA levels, however, were not higher in the better differentiated group and there were not significantly more pNR-2 negative tumours in the poorly differentiated group. The finding that seven grade I/II tumours were pNR-2 negative while none were oestrogen receptor mRNA negative was of interest as it might suggest that the capacity to express pNR-2 is lost at an earlier stage in tumour de-differentiation.

There was a clear and highly significant association between previous response to tamoxifen treatment and the presence of pNR-2 mRNA in tumours of patients receiving tamoxifen as primary endocrine therapy. Although the measurements of pNR-2 were made following disease progression, this finding has implications for the value of pNR-2 as a predictive marker of response to tamoxifen in primary breast cancer.

In addition, the high levels of pNR-2 mRNA expression in tumours whose growth had been but is no longer inhibited by tamoxifen has important implications for models of tamoxifen resistance. The tumour levels of both oestrogen receptor and pNR-2 mRNAs were no different in the patients who had responded to and then relapsed on tamoxifen than found in patients who had not received primary tamoxifen therapy. This suggests that, in the majority of cases, relapse is not associated with the outgrowth of oestrogen receptor negative cells that are unresponsive to tamoxifen. Experiments with tamoxifen-resistant oestrogen-responsive cell lines have shown that tamoxifen resistance may be associated with an increased agonist effect of tamoxifen on cell proliferation (M.D. Johnson *et al.*, in preparation) and these data from patients receiving primary tamoxifen therapy are consistent with this.

Two tumours did not contain oestrogen receptor mRNA but had responded to tamoxifen. Their response is difficult to rationalise but it is possible that these two tumours ceased to respond to tamoxifen as a result of the outgrowth of oestrogen receptor negative cells.

There would be considerable clinical advantage in accurate prediction of response to primary tamoxifen therapy using the small amount of material provided by non-surgical diagnostic techniques such as fine needle aspiration or needle biopsy. mRNA amplification techniques or immunohistochemical staining for the pNR-2 protein product may permit assay in such material, allowing assessment of pNR-2 as a marker of anti-oestrogen response.

This work was supported by the North of England Cancer Research Fund. We thank Professor P. Chambon for the oestrogen receptor cDNA. J. A. Henry thanks the Wellcome Trust for a research training fellowship. F.E.B. May is a recipient of a 1983 University Research Fellowship from the Royal Society.

### References

BERGER, M.S., LOCHER, G.W., SAURER, S. & 4 others (1988). Correlation of c-*erb* B-2 gene amplification and protein expression in human breast cancer with nodal status and nuclear grading. *Cancer Res.* **48**, 1238.

BLOOM, H.J.G. & RICHARDSON, W.W. (1957). Histological grading and prognosis in breast cancer: a study of 1409 cases of which 359 have been followed for 15 years. *Br. J. Cancer*, **11**, 359.

DARBRE, P.D., CURTIS, S. & KING, R.J.B. (1984). Effects of estradiol and tamoxifen on human breast cancer cells in serum-free culture. *Cancer Res.*, **44**, 2790.

DESOMBRE, E.R., GREENE, G.L. & JENSEN, E.V. (1980). Estrogen receptors and hormone dependence of breast cancer. In *Breast Cancer: New Concepts in Etiology and Control*, Brennen, M.J., McGrath, C.M. & Rich, M.A. (eds) p. 69. Academic Press: New York.

ENGEL, W.L., YOUNG, N.A., TRALKA, T.S., LIPPMAN, M.E., O'BRIEN, S.J. & JOYCE, M.J. (1978). Establishment and characterization of three new continuous cell lines derived from human breast carcinomas. *Cancer Res.*, **38**, 3352.

HENRY, J.A., NICHOLSON, S., FARNDON, J.R., WESTLEY, B.R. & MAY, F.E.B. (1988). Measurement of oestrogen receptor mRNA levels in human breast tumours. *Br. J. Cancer*, **58**, 600.

JAKOWLEW, S.B., BREATHNACH, R., JELTSCH, J.-M., MASIAKOWSKI, P. & CHAMBON, P. (1984). Sequence of the pS2 mRNA induced by estrogen in the human breast cancer cell line MCF-7. *Nucleic Acids Res.*, **12**, 2861.

LIPPMAN, M.E. & BOLAN, G. (1975). Oestrogen-responsive human breast cancer in long term tissue culture. *Nature*, **256**, 592.

McCARTY, K.S. BARTON, T.K. & FETTER, B.F. (1980). Correlation of estrogen and progesterone receptors with histological differentiation in mammary carcinoma. *Cancer*, **46**, 2851.

McGUIRE, W.L., CARBONNE, P.D. & VOLLMER, R.P. (eds) (1975). *Estrogen Receptor and Human Breast Cancer*. Raven Press: New York.

MASIAKOWSKI, P., BREATHNACH, R., BLOCH, J., GANNON, F., KRUST, A. & CHAMBON, P. (1982). Cloning of cDNA sequences of hormone regulated genes from the MCF-7 human breast cancer cell line. *Nucleic Acids Res.*, **10**, 7895.

MAY, F.E.B. & WESTLEY, B.R. (1986). Cloning of estrogen regulated messenger RNA sequences from human breast cancer cells. *Cancer Res.*, **46**, 6034.

MAY, F.E.B. & WESTLEY, B.R. (1987). Effects of tamoxifen and 4-hydroxytamoxifen on the pNR-1 and pNR-2 estrogen-regulated RNAs in human breast cancer cells. *J. Biol. Chem.*, **262**, 15894.

MAY, F.E.B. & WESTLEY, B.R. (1988). Identification and characterisation of oestrogen regulated RNAs in human breast cancer cells. *J. Biol. Chem.*, **263**, 12901.

MELTON, D.A., KREIG, P.A., REBAGLIATI, M.R., MANAITIS, T., ZINN, K. & GREEN, M.R. (1984). Efficient in vitro synthesis of biologically active RNA and RNA hybridisation probes from plasmids containing a bacteriophage SP6 promoter. *Nucleic Acids Res.*, **12**, 7035.

NUNEZ, A.-M., JAKOWLEV, S., BRIAND, J.-P. & 4 others (1987). Characterisation of the estrogen-induced pS2 protein secreted by the human breast cancer cell line MCF-7. *Endocrinology*, **121**, 1759.

OSBORNE, C.K., YACHINOWIZ, M.G., KNIGHT, W.A. & McGUIRE, W.L. (1980). The value of estrogen and progesterone receptors in the treatment of breast cancer. *Cancer*, **46**, 2884.

PRUD' HOMME, J.-F., FRIDLANSKY, F., LE CUNFF, M. & 4 others (1985). Cloning of a gene expressed in human breast cancer and regulated by estrogen in MCF-7 cells. *DNA*, **4**, 11.

RIO, M.C., BELLOCQ, J.P., GAIRARD, B. & 7 others (1987). Specific expression of the pS2 gene in subclasses of breast cancers in comparison with expression of the estrogen and progesterone receptors and the oncogene ERBB2. *Proc. Natl Acad. Sci. USA*, **84**, 9243.

RIO, M.C., BELLOCQ, J.P., DANIEL, J.Y. & 5 others (1988). Breast cancer associated pS2 protein: synthesis and secretion by normal stomach mucosa. *Science*, **241**, 705.

SOULE, H.D., VASQUEZ, J., LANG, A., ALBERT, S. & BRENNAN, M.A. (1973). A human cell line from a pleural effusion derived from a breast carcinoma. *J. Natl Cancer Inst.*, **51**, 1409.

SOUTHERN, E.M. (1975). Detection of specific sequences among DNA fragments separated by gel electrophoresis. *J. Molec. Biol.*, **98**, 503.

WALTER, P., GREEN, S., GREENE, G. & 8 others (1985). Cloning of the human estrogen receptor cDNA. *Proc. Natl Acad. Sci. USA*, **82**, 7889.

WESTLEY, B.R. & MAY, F.E.B. (1984). The human genome contains multiple sequences of varying homology to mouse mammary tumour virus DNA. *Gene*, **28**, 221.

WESTLEY, B.R. & MAY, F.E.B. (1987). Oestrogen regulates cathepsin D mRNA levels in oestrogen responsive human breast cancer cells. *Nucleic Acids Res.*, **15**, 3773.

WESTLEY, B.R., HOLZEL, F. & MAY, F.E.B. (1989). Effects of oestrogen and the antioestrogens, tamoxifen and LY117018, on four oestrogen regulated RNAs in the EFM-19 breast cancer cell line. *J. Steroid Biochem.*, **32**, 365.

ZAJCHOWSKI, D., BAND, V., PAUZIE, N., TAGER, A., STAMPFER, M. & SAGER, R. (1988). Expression of growth factors and oncogenes in normal and tumour-derived human mammary epithelial cells. *Cancer Res.*, **48**, 7041.